# Enhanced Energy Transfer from a Metal–Organic Framework to a Highly Confined Organic Phosphorescent Dye

**DOI:** 10.1002/advs.202600068

**Published:** 2026-03-15

**Authors:** Bahram Hosseini Monjezi, Thomas Kasper, Gloria Hong, Changfeng Si, Robert Oestreich, Oliver Weingart, Peter G. Weidler, Christof Wöll, Eli Zysman‐Colman, Christoph Janiak, Stefan Bräse, Klaus Müller‐Buschbaum, Alexander Knebel

**Affiliations:** ^1^ Institute of Functional Interfaces (IFG) Karlsruhe Institute of Technology (KIT) Karlsruhe Germany; ^2^ Institute for Nuclear Waste Disposal (INE) Karlsruhe Institute of Technology (KIT) Karlsruhe Germany; ^3^ Institute of Inorganic and Analytical Chemistry Justus Liebig University Giessen Giessen Germany; ^4^ Institute of Organic Chemistry (IOC) Karlsruhe Institute of Technology (KIT) Karlsruhe Germany; ^5^ Organic Semiconductor Centre EaStCHEM School of Chemistry University of St Andrews St Andrews UK; ^6^ Institute of Inorganic and Structural Chemistry Heinrich Heine University Düsseldorf Düsseldorf Germany; ^7^ Institute of Theoretical and Computational Chemistry Heinrich Heine University Düsseldorf Düsseldorf Germany; ^8^ Institute of Biological and Chemical Systems – Functional Molecular Systems (IBCS‐FMS) Karlsruhe Institute of Technology (KIT) Karlsruhe Germany; ^9^ Center of Materials Research (LAMA) Justus Liebig University Giessen Giessen Germany; ^10^ Otto Schott Institute for Materials Research Center of Energy and Environmental Chemistry II Friedrich Schiller University Jena Jena Germany

**Keywords:** aggregation control, energy transfer, host‐guest interaction, metal–organic frameworks, phosphorescence, thin films

## Abstract

Three‐Dimensional Metal–Organic Frameworks (MOFs) are crystalline, porous hybrid materials with well‐defined pore structures, offering isotropic adsorption of molecules in their pore system. We designed and synthesized an organic, phosphorescent di‐*tert*‐butyl‐carbazole dibenzophenazine chromophore, **11‐DTCz‐BP**, with an appropriate size for a tight hand‐to‐glove fit into the 1D pore channels of MIL‐68(In). MOF particles are synthesized, and homogeneous films are prepared on Au‐substrates through electrochemical deposition. The steric demand of **11‐DTz‐BP** yields a crystallographic ordered confinement inside the pores of MIL‐68(In), proven by XRD and DFT calculations. Through this approach, it was possible to obtain controlled packing of the chromophore and lower emission quenching factors. Detailed spectroscopic analysis was performed using (cryo)fluorescence spectroscopy on powders and thin films. We consider Förster Resonance Energy Transfer (FRET) as a process of energy transfer between the pore walls of MIL‐68 and **11‐DTCz‐BP**. The aggregation control leads to a quantum yield enhancement, while FRET enables long lifetimes of phosphorescence at room‐temperature.

## Graphical abstract

A phosphorescent dye was designed to fit in a hand‐to‐glove fashion into the 1D‐pore channels of MIL‐68(In). We synthesized MIL‐68(In) as powder and thin films, loaded the dye into the pores with controlled packing, and found that the phosphorescence and quantum yield are enhanced. A highlight of this material is the room‐temperature phosphorescence lifetime, which is highly elongated.

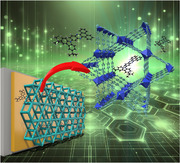

## 1 Introduction

Metal–Organic Frameworks (MOFs) have garnered significant attention in recent years due to their versatile applications, including adsorptive gas storage [1], molecular sieving separations [2, 3], (photo)catalysis [4], and their role as battery materials [5] and sensors [6]. Of particular importance in this study is their function as optical active materials. MOFs are porous, crystalline structures formed through coordinative bonds between metal centers or clusters (Secondary Building Unit or SBU) and organic linkers. This results in a wide range of reticular materials with diverse chemical and physical characteristics [7]. Chemical functionalization allows for the alteration of pore dimensions and properties. The linker and SBU both play a crucial role in energy transfer processes in luminescent MOFs, for instance, through open metal‐sides and free electron or via electron donor–acceptor and antenna effects [7, 8].

Energy transfer processes in luminescent MOFs primarily involve metal‐to‐metal, metal‐to‐ligand, or antenna‐like linker‐to‐metal electronic coupling [8, 9]. Although less common, linker‐to‐linker energy transfer can also occur. Molecular rotors embedded in MOFs have demonstrated functionality by visible light‐induced linker‐to‐linker energy transfer [10].

Various photonic MOF materials have been synthesized using lanthanoid centers. The controlled layer‐by‐layer synthesis of surface‐anchored MOF thin films (SURMOFs) has, for instance, enabled the formation of a white light‐emitting film [11]. Additionally, incorporating powders of Ln‐MOFs into polymer matrices allowed for humidity sensing, utilizing emission quenching upon contact with water [12]. This humidity sensing mechanism, in which acetone vapors induce a luminescence response in MOFs, serves as an effective medical sensor [13].

Guest molecules with strong and stable luminescent properties can provide reliable optical responses [14]; however, without controlled chromophore aggregation, undesirable effects such as quenching may occur, which can significantly reduce luminescence efficiency. Therefore, precise aggregation control is crucial for developing highly potent luminescent materials [15]. This is particularly important when studying solvated, non‐luminescent thermally delayed activated fluorescent chromophores [16]. When incorporated into a crystalline MOF backbone, these chromophores enable MOF thin films to exhibit both prompt luminescence and electroluminescence in OLEDs [17]. Another key area of interest is room‐temperature phosphorescence, which has broad applications. Controlling molecular coordination within MOF pores while preventing aggregation can help regulate phosphorescence behavior, either following the Kasha rule or stabilizing desired anti‐Kasha phosphorescence [18, 19].

In 1D pore channels, chromophore aggregation is naturally controlled [20]. We propose that this aggregation follows an isotropic packing order with a specific energy minimum, other than in 3D MOFs, where guest orientation is often less constrained and anisotropic ordering of dyes is found [21]. This differs from cage‐type pore systems with higher dimensionality, which have been described in terms of “static porosity” versus “dynamic porosity,” a concept we leverage here [22]. Effective entrapment of guest molecules can enhance host–guest interactions, optimize aggregation behavior of luminescent guests, and enable energy transfer from the lattice to the guest molecules [23, 24]. However, in many reported MOF–guest phosphorescent systems, the guest is selected from known luminophores and incorporated by non‐specific loading, which can limit control over guest orientation/packing and often restrict loading to avoid quenching. In this work, we adopt an a priori host–guest design strategy: the phosphorescent dye 11‐DTCz‐BP was custom‐synthesized to be geometrically commensurate with the MOF channels, aiming to suppress aggregation‐driven quenching at higher loading and to promote efficient host–guest coupling.

Considering this, selecting an appropriate MOF is crucial as its inorganic building unit should be a suitable sensitizer to transfer absorbed energy. [25] Consequently, our investigation focused on the indium‐based Kagome‐type MOF [In(OH)bdc] (bdc = benzene‐1,4‐dicarboxylate, terephthalate), also known as MIL‐68(In) (Matériaux de l'Institut Lavoisier) [26, 27]. MIL‐68(In) crystallizes in an orthorhombic crystal system with the space group *Cmcm*, featuring two types of 1D channel openings: ∼6 Å (trigonal) and ∼16 Å (hexagonal), with an estimated variation of ± 2 Å due to rotational flexibility of the benzene linkers (gate opening). These well‐defined pore sizes facilitate the spatial confinement of guest molecules. This is also a structure proposed for static porosity [22] and thereby could control chromophore packing in the channels through a pore system fitting like a glove.

To achieve any kind of energy transfer between host and guest, one approach is Förster resonance energy transfer (FRET) through a size‐matched confinement of the chromophore within the pore channels [28]. While other groups have demonstrated phosphorescence and thermally activated delayed fluorescence (TADF) by encapsulating small quantities (0.2%–1.5%) of chromophores and non‐specific loading, which can limit control over guest orientation/packing that often restrict loading [29, 30], our approach involves a priori design approach, synthesizing a phosphorescent dye tailored to the 1D channel geometry of MIL‐68(In), enabling high loading while suppressing aggregation‐driven quenching and yielding enhanced PLQY and long‐lived room‐temperature emission [31]. Consistent with this confinement strategy, the composite shows a measurable improvement in photophysical performance (increased PLQY and prolonged room‐temperature emission lifetime) compared with the free dye.

## 2 Results and Discussion

**FIGURE 1:**
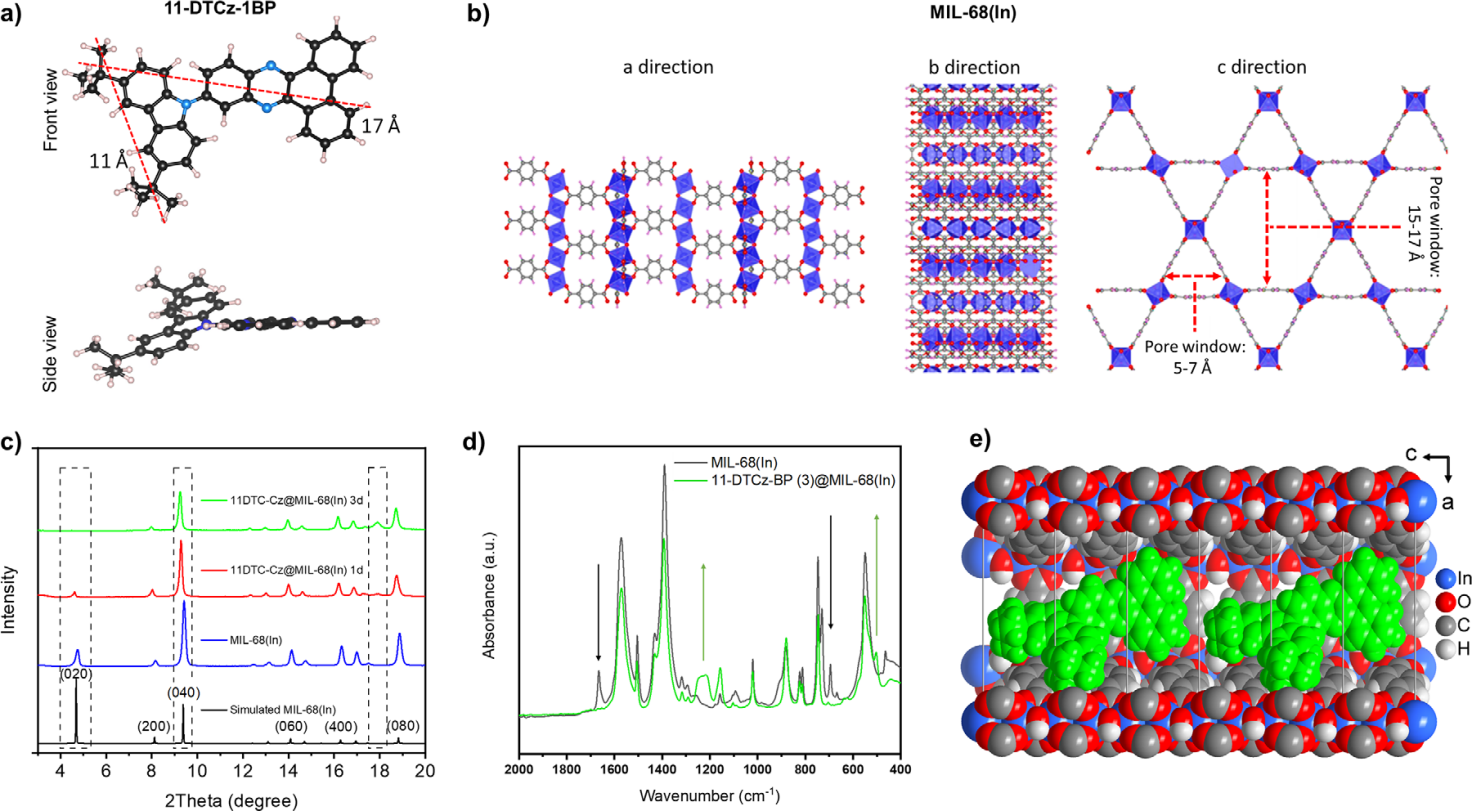
(a) The molecular structure and dimensions of the phosphorescent 11‐DTCz‐1BP dye. (b) The crystal structure of MIL‐68(In) in a‐, b‐, and c‐directions, with the Kagome structure visible in c‐direction, depicting hexagonal (∼16 Å) and triangular (∼6 Å) 1‐D channels [35]. (c) The PXRD analysis of loading MIL‐68(In) powder with 11‐DTCz‐1BP from solution to form 11‐DTCz‐1BP@MIL‐68(In) over the course of 3 days, showing crystallographic changes. (d) FTIR spectra of MIL‐68(In) and 11‐DTCz‐1BP@MIL‐68(In) showing significant changes to vibrational modes. (e) Simulation of the pore fitting of 11‐DTCz‐1BP along the 1D pore channels depicted for the b‐direction of MIL‐68(In). The vertical grey lines are the unit cell edges along the c direction, thus showing six‐unit cells.

The chromophore **11‐DTCz‐BP** (11‐(3,6‐di‐*tert*‐butyl‐9*H*‐carbazol‐9‐yl)dibenzo[*a*,*c*]phenazine) was developed especially for this study based on simulations of its energy levels and spectroscopic properties (Figure 1a and Supporting Information). The electron‐donating 3,6‐di‐*tert*‐butyl‐9*H*‐carbazole (DTCz) unit and the electron‐accepting dibenzo[*a*,c]phenazine (BP) moiety are twisted with respect to each other with a dihedral angle of 48.8°. Thus, the frontier molecular orbitals are well‐separated. The HOMO‐LUMO energy gap is 3.23 eV with a HOMO at ‐5.48 eV and a LUMO at ‐2.25 eV (Figure S8). The excited‐state properties were calculated using time‐dependent density functional theory (TD‐DFT) within the Tamm‐Dancoff approximation (TDA) based on the ground‐state optimized geometry at the same level of theory. A comparatively large singlet–triplet energy gap (Δ*E*_ST_ = 0.38 eV) was calculated, indicating the chromophore's potential for efficient energy transfer and suitability for luminescent applications.

The MOF MIL‐68(In) was prepared as a powder using conventional solvothermal synthesis, and a thin film on Au‐substrates through electrochemical deposition (SI). MIL‐68(In) exhibits a Kagome lattice with indium (In^3+^) metal‐nodes, interconnected by terephthalate linkers. The channels with accessibility for the chromophore are 1D and lie in the c‐direction (Figure 1b). The size of the chromophore is crucial to achieve the highest chromophore loading, yet also prevent leeching and quenching, and so was adjusted to be just slightly smaller than the window size of the large hexagonal channels in the MOF. Figure 1c demonstrates the loading of a powder sample of MIL‐68(In) with **11‐DTCz‐BP** over the course of three days to form the composite **11‐DTCz‐BP@MIL‐68(In**), as evidenced by powder X‐ray diffraction (PXRD) of the powder. Upon dye incorporation into the MOF, the reflection of the MOF at 2θ = 4.7 °2θ gradually becomes less intense, while a new reflection emerges at 17.9 °2θ. In addition, the reflection at 2θ ≈ 9.4° shifts slightly to lower angles, consistent with a modest framework expansion upon guest inclusion. These changes indicate guest‐induced modulation of the diffraction pattern while preserving the overall crystallinity of MIL‐68(In). The appearance of the additional reflection at 2θ = 17.9°, which is absent in the pristine framework, suggests non‐random incorporation of the dye molecules within the 1D channels, although full crystallographic refinement of the guest arrangement is beyond the scope of laboratory PXRD. Similar guest‐induced changes in peak intensity and position have been reported for other MOF–dye systems and are commonly attributed to electron‐density redistribution and preferred guest–framework interactions [32]. To further assess the structural consistency of the proposed host–guest model, a simulated PXRD pattern based on the DFT‐optimized structure shown in Figure 1e is provided in SI (Figure S9). The simulated pattern reproduces the emergence of additional reflections and subtle peak shifts associated with guest inclusion and framework expansion. In our case, the attenuation of the 4.7 °2θ reflection is attributed to these effects, while the overall crystalline structure of the MOF remains well‐preserved [33]. The FT‐IR spectrum of **MIL‐68(In)** shows bands at 1600–1400 cm^−1^ that are characteristic of the aromatic compounds, along with stretching vibrations of the ‐COOH groups (Figure 1d). Specifically, the bands at 1572, 1430, and 1400 cm^−1^ are attributed to C─C stretching vibrations of integral benzene rings. After loading of **11‐DTCz‐BP**, all sharp peaks originating from **MIL‐68(In)** remained, which reveals that the integrity of the **MIL‐68(In)** structure is preserved throughout the guest incorporation process [34]. In addition, the appearance of new absorption bands in the FTIR spectrum at 1250, 1320, and 1666 cm^−1^ is attributed to the C–N stretching vibrations of **11‐DTCz‐BP** guest.

We complemented the experimental study by investigating the **11‐DTCz‐BP** adsorption and pore fitting using DFT methods (Figure 1e, and Supporting Information). With the content of an asymmetric unit being [In_3_(OH)_3_(bdc)_3_] and Z = 4, the unit cell comprises [In_12_(OH)_12_(bdc)_12_]. There are two hexagonal channels per unit cell, which each can be filled with 1/3 of an **11‐DTCz‐BP** molecule, and thus giving a maximum pore filling of (**11‐DTCz‐BP**)**_2/3_@[In_12_(OH)_12_(bdc)_12_]** or (**11‐DTCz‐BP**)**_1/18_@[In(OH)bdc]**.

**FIGURE 2:**
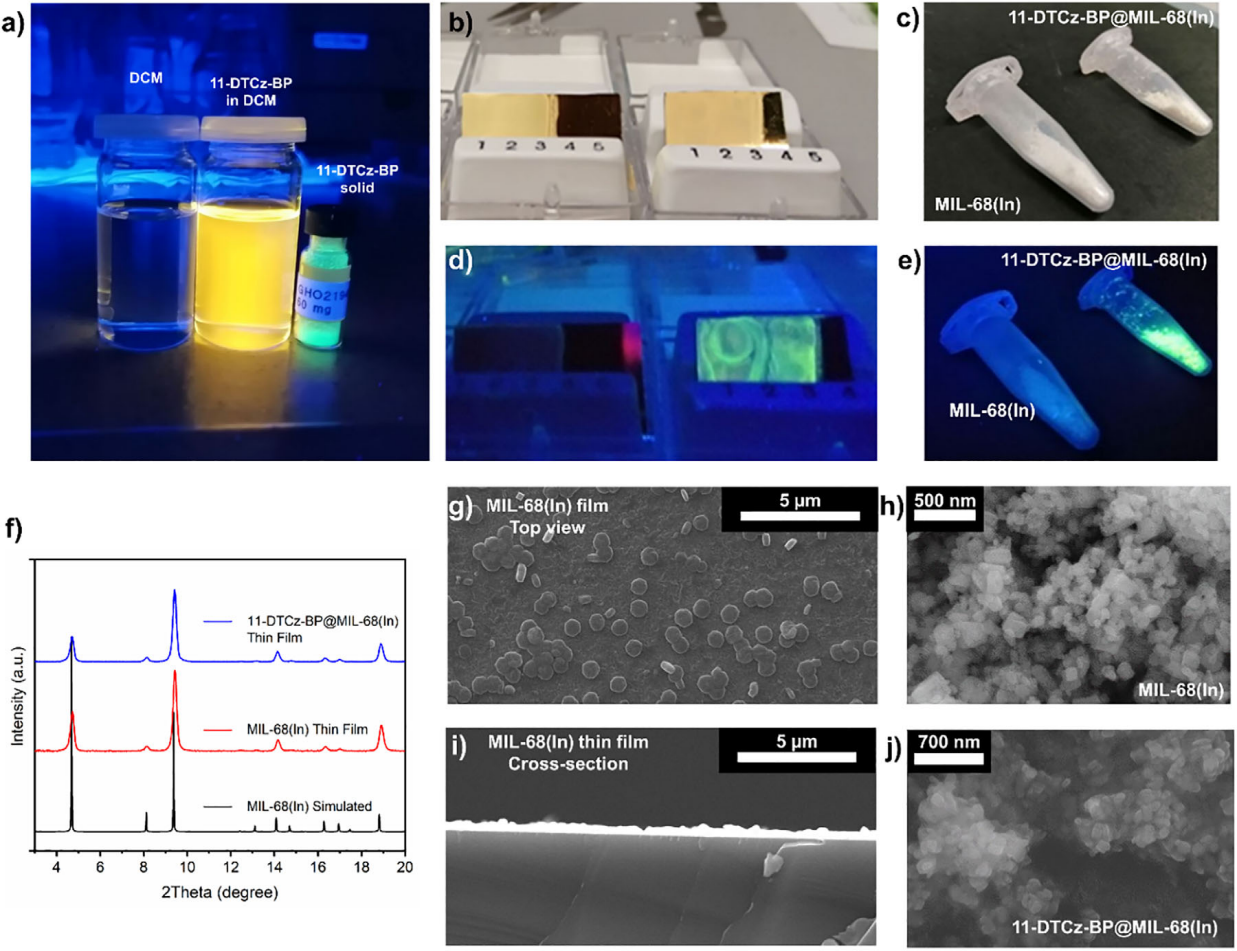
(a) A photograph of DCM, 11‐DTCz‐BP in DCM, and the solid 11‐DTCz‐BP luminescence under UV‐light. (b) Photograph of electrochemically synthesized thin films of MIL‐68(In) and 11‐DTCz‐BP@MIL‐68(In). (c) Photograph of MIL‐68(In) and 11‐DTCz‐BP@MIL‐68(In) powders in vials. (d) Photograph of thin films of MIL‐68(In) and 11‐DTCz‐BP@MIL‐68(In) under UV‐light showing luminescence. (e) Photograph of MIL‐68(In) and 11‐DTCz‐BP@MIL‐68(In) powders in vials under UV‐light showing luminescence. (f) XRDs of the MIL‐68(In) and 11‐DTCz‐BP@MIL‐68(In) thin films showing comparable results to the powder loading in Figure 1c). (g) SEM image of the MIL‐68(In) thin film (h) SEM image of the MIL‐68(In) powder. (i) Cross‐section of the MIL‐68(In) thin film. (j) SEM image of the 11‐DTCz‐BP@MIL‐68(In) powder, showing no morphological changes.

**11‐DTCz‐BP** displays yellow emission in dichloromethane (DCM), likely due to solvatochromism in the polar solvent, while its solid‐state luminescence appears green (Figure 2a). The calculated binding energy of **11‐DTCz‐BP** to the pore wall is 129 kJ/mol, due to strong confinement. The **MIL‐68(In)** thin film in Figure 2b is synthesized through electrochemical deposition, which is a fast way to produce a functional layer, and has been subsequently loaded with **11‐DTCz‐BP**. The neat MIL‐68(In) and loaded **11‐DTCz‐BP**@MIL‐68(In) powder samples are shown in Figure 2c. The green luminescence of the powder and the thin film is depicted in the photograph (Figure 2d,e). The PXRD of the film shows the same XRD as that of the simulated sample, meaning that the film is similarly polycrystalline. The crystalline ordering of the dye cannot be seen in the PXRD measurement, probably due to the very small dimension of the thin film; however, the peak intensity of the first peak changed upon **11‐DTCz‐BP**@MIL‐68(In) loading, which is likewise consistent with the adsorption experiments of the powder sample. The scanning electron microscopy (SEM) analysis is shown in Figure 2g–j.

The SEM images shown in Figure 2g,h are of the pristine film and pristine powder samples, respectively, while Figure 2i shows the cross‐section of the MIL‐68(In) thin film with a thickness of 500 nm, while Figure 2j presents composite powder **11‐DTCz‐BP**@MIL‐68(In) with no observable changes besides sample conductivity [36] and thus, contrast in the electron microscope image. The thermal stability and dye loading of **MIL‐68(In)** and **11‐DTCz‐BP@MIL‐68(In)** were assessed using thermogravimetric analysis (TGA), and the detailed data are provided in the Supporting Information. Both samples possess similar thermal stability, remaining intact up to 400°C. The reduced final mass after thermal decomposition for **11‐DTCz‐BP**@MIL‐68(In) was lower (30.1%) compared to MIL‐68(In) (42.7%), indicating successful incorporation of the dye into the pores. Based on the residual mass difference, the dye loading was estimated at approximately 12 wt.%.

**FIGURE 3:**
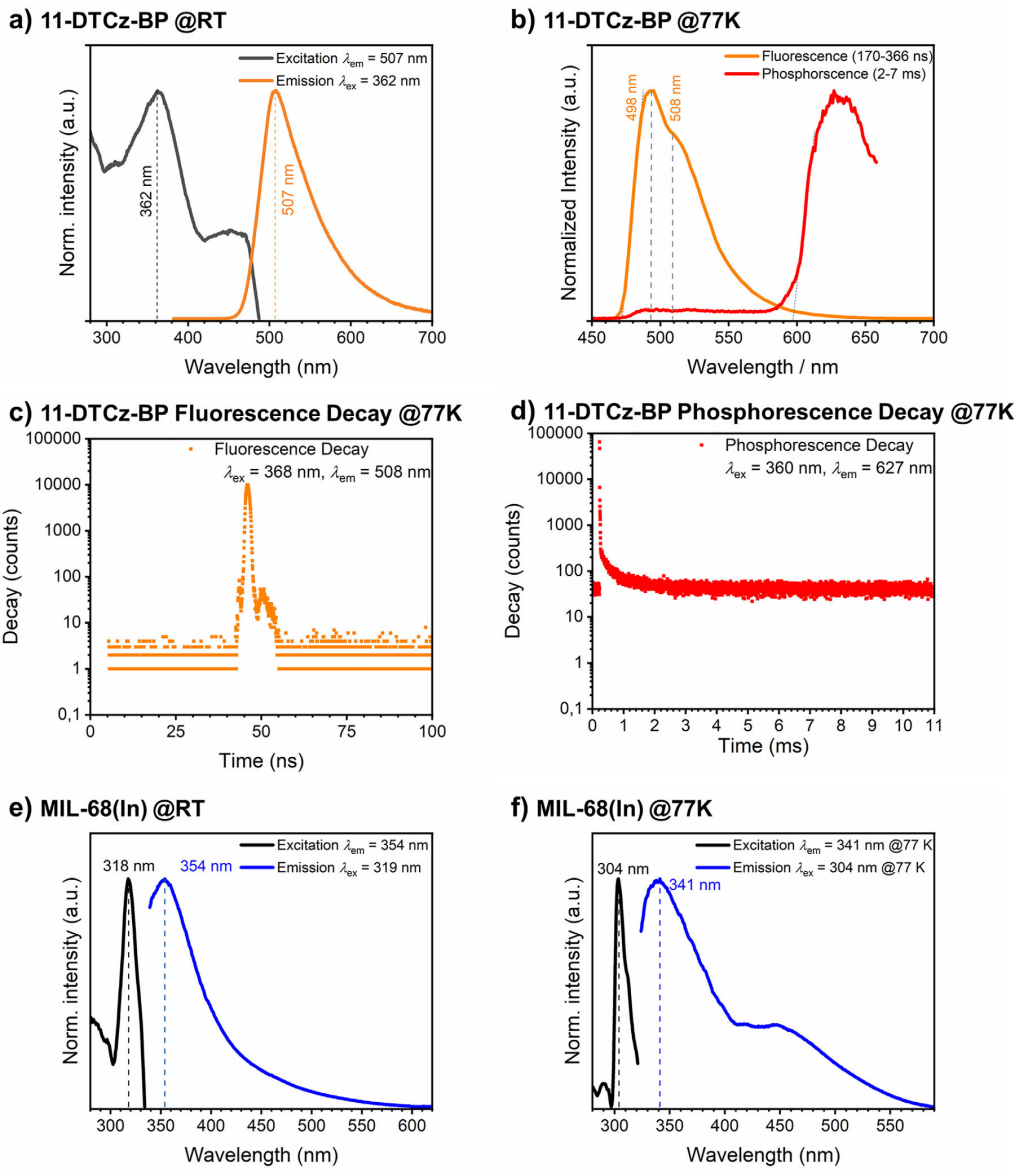
(a) Excitation and fluorescence emission spectrum of solid 11‐DTCz‐BP at room‐temperature (RT = 293 K). (b) Fluorescence and phosphorescence emission spectrum of solid 11‐DTCz‐BP at 77 K, (c) 11‐DTCz‐BP fluorescence decay at 77 K. (d) 11‐DTCz‐BP phosphorescence decay at 77 K. (e) Excitation and emission spectrum of MIL‐68 (In) at 293 K. (f) Excitation and emission spectrum of MIL‐68(In) at 77 K.

An initial photophysical investigation is provided in Figure 3. The excitation and emission spectra of **11‐DTCz‐BP** in the solid‐state at room‐temperature (RT) show an emission peaking at 507 nm (Figure 3a), reflective of the observed green emission in the solid‐state. The **11‐DTCz‐BP** appears yellow in dichloromethane solution (Figure 2a). Several environmental parameters, such as polarity, viscosity, and hydrogen bonding interactions, have an impact on the charge‐transfer process of excited molecules, which is the reason why the luminescence of **11‐DTCz‐BP** is different when dissolved in DCM (see Supporting Information) [37]. However, a reasonable explanation for this observation could also be an agglomeration of the dye molecules in solution, which can lead to altered intermolecular interactions, enhanced non‐radiative energy transfer, and concentration quenching, thereby impacting the emission characteristics.

Since the calculated energy difference of the **11‐DTCz‐BP** molecule between the lowest triplet (E_T_ = 2.29 eV) and singlet (E_S_ = 2.68 eV) excited states (Δ*E*_ST_) is small and exhibits a band at 507 nm, it can be attributed to prompt fluorescence. The photoluminescence spectrum of **11‐DTCz‐BP** at 77 K in the solid‐state shows an emission wavelength maximum at 498 nm and a small bump at 508 nm, with a phosphorescence peaking at 632 nm (see Figure 3b).

The time‐resolved PL decays at different time windows are given in Figure 3c,d. At 77 K, the overall process decay of the fluorescence is as expected, very short at around 10 ns, while the phosphorescence decay is about 0.3 ms. The MOF itself displays weak emission, and the PL spectrum of MIL‐68(In) is given in Figure 3e, f for measurements at 293 and 77 K. The two decay components observed for the MIL‐68(In) at 77 K are assumed to be from two different and temperature‐dependent occupations of the excited states. Additionally, there is a blue shift in the emission spectrum of 13 nm from 354 nm at 293 K to 341 nm at 77 K and a hypsochromic shift of 14 nm from 318 nm at 293 K to 304 nm at 77 K in the excitation spectrum as shown in Figure 3e,f, which we assume to be a temperature‐dependent charge‐transfer effect. This gives a spectral overlap between the emission spectrum of MOF and the excitation/absorption spectrum of **11‐DTCz‐BP**, allowing for an emission‐absorption energy transfer. However, further investigation reveals that this interaction is predominantly governed by FRET rather than simple emission‐absorption energy transfer [38].

**FIGURE 4:**
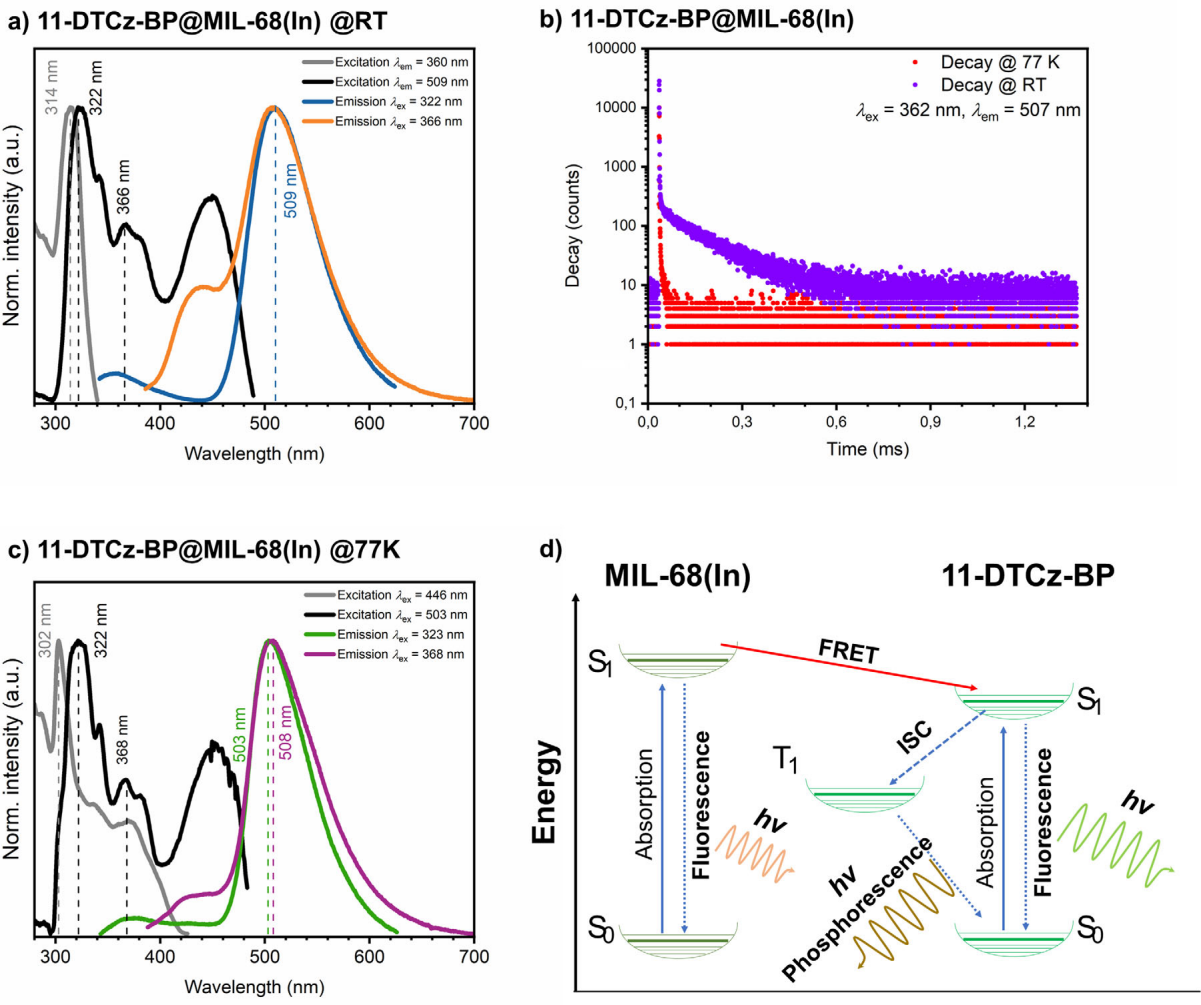
(a) Excitation and emission spectra of the 11‐DTCz‐BP@MIL‐68(In) composite at four different wavelengths. (b) Phosphorescent decay of 11‐DTCz‐BP@MIL‐68(In) with a surprising phosphorescent lifetime elongation at 293 K compared to a decay at 77 K, comparable to the phosphorescence decay of the dye alone (Figure 3d). (c) Excitation and emission spectra of the 11‐DTCz‐BP@MIL‐68(In) composite at 77 K. (d) A schematic of the energy transfer driven by FRET between MIL‐68(In) and 11‐DTCz‐BP.

The emission spectra of **11‐DTCz‐BP**@MIL‐68(In) at RT are given in Figure 4a. A strong increase in the overall lifetime at RT when **11‐DTCz‐BP** is incorporated in **11‐DTCz‐BP**@MIL‐68(In) is measured, while the overall decay time at 77 K, which equals the phosphorescence lifetime of the pure **11‐DTCz‐BP** at 77 K, as shown in Figure 4b. The change of the surrounding media blue‐shifts the wavelength of phosphorescence. We assume that a locally exciting state of the MIL‐68(In) and the linker (donor) leads to a strong energy transfer onto **11‐DTCz‐BP** (acceptor). We attribute this to the packing control of **11‐DTCz‐BP** in the 1D pores of MIL‐68(In) by the near hand‐to‐glove fit of the molecular dimension, which is allowing for triplet‐to‐singlet FRET. To assess whether the designed host–guest confinement leads to a measurable functional advantage beyond simple dye encapsulation, we analyzed both photoluminescence quantum yields (PLQY) and excited‐state lifetimes. Time‐resolved measurements reveal a qualitative change in the excited‐state dynamics upon confinement. As summarized in Table S1, the free 11‐DTCz‐BP molecule exhibits only nanosecond‐scale decay at room‐temperature, whereas the 11‐DTCz‐BP@MIL‐68(In) composite shows prolonged excited‐state lifetimes and the emergence of a microsecond‐scale emission component that is absent in the free dye. This stabilization of long‐lived emission at room‐temperature highlights the functional impact of host–guest coupling and confinement within the 1D channels of MIL‐68(In).

Additionally, in Table S2, the PLQY of 11‐DTCz‐BP@MIL‐68(In) reaches 13.8%, representing an approximately 50% increase compared to the free 11‐DTCz‐BP (9.0%), while the host framework alone exhibits a much lower PLQY (∼3.3%). This non‐additive enhancement indicates that confinement within the MIL‐68(In) channels effectively suppresses non‐radiative decay pathways, consistent with reduced aggregation‐induced quenching. When considered together with the increase in photoluminescence quantum yield (Table S2), these results demonstrate that the size‐matched confinement strategy provides a clear photophysical advantage over non‐optimized or randomly loaded dye systems, without requiring full crystallographic ordering of the guest molecules.

From the literature, triplet‐to‐singlet FRET (TS‐FRET), which is also called phosphorescence resonance energy transfer (PRET), involves energy transfer from an excited triplet state (donor) to an excited singlet state (acceptor) [39]. The energy transfer that is happening here could potentially be TS‐FRET, and this process plays an important role in constructing high‐efficiency phosphorescent materials [40]. FRET is a dipole–dipole coupled process, which depends on the distance of the excited and ground state species. Our system closes the distance between the MOF pore walls and the dipolar **11‐DTCz‐BP** molecule and observes spectral broadening, which could be an indication for FRET. FRET is also known to be temperature dependent, which we think leads to a reasonable increase of energy transfer at 293 K compared to 77 K, as the compounds have a different distance at different temperatures and energy levels shift in temperature dependence (c.f. simulations in Supporting Information). Construction of RT phosphorescent materials by the effect of tight stacking of phosphorescent molecules has been demonstrated via FRET [41]. By itself, **11‐DTCz‐BP** shows a comparably short phosphorescence lifetime at both temperatures of less than 0.1 ms, respectively (see Figure 3d). The proposed mechanism for the energy transfer between MOF and **11‐DTCz‐BP** is given in Figure 4d. The phosphorescence decay is longer than for pure **11‐DTCz‐BP** (c.f. Figure 3d) by about 0.5 ms.

## 3 Conclusion

In summary, we demonstrated in this study the successful design and synthesis of a phosphorescent organic chromophore, **11‐DTz‐BP**, that fits precisely within the 1D pore channels of MIL‐68(In). This precise hand‐to‐glove fit allows for effective control over the chromophore's aggregation behavior by ordered packing, which reduces emission quenching and enhances the quantum yield of the system. This was demonstrated on powders of MIL‐68(In) incorporated with **11‐DTz‐BP** and **11‐DTz‐BP** @MIL‐68(In) and **11‐DTz‐BP** @MIL‐68(In) thin films made on gold substrates (see Supporting Information). The successful formation of **11‐DTz‐BP**@MIL‐68 was validated using powder X‐ray diffraction and density functional theory calculations. Fluorescence spectroscopy analysis supports Förster Resonance Energy Transfer (FRET) between the MOF pore walls and **11‐DTz‐BP**. Exciting the MOF leads to a donor–acceptor energy transfer, resulting in prolonged phosphorescence lifetimes not visible at 77 K, but surprisingly at room‐temperature at 293 K. The thermal expansion and mobility of the MOF toward the **11‐DTz‐BP** at RT seem to be crucial. This approach of tightly fitting a chromophore into the 1D pore channels of MOFs demonstrates the potential of MOFs for controlled chromophore confinement, enhancing the optical properties of both materials. To summarize the results in an outlook, we found a very useful way to control aggregation and agglomeration behavior of chromophores by low‐dimensional pore systems, while enhancing photoluminescent quantum yield and phosphorescence lifetimes, which could find potential application in organic light‐emitting diodes. While this study primarily focuses on the enhanced photoluminescent behavior resulting from host–guest interactions in **11‐DTCz‐BP**@MIL‐68(In), the structural versatility and confined molecular arrangement within the MOF suggest strong potential for additional functional applications such as chemical sensing or charge transport, which will be the subject of future investigations. Furthermore, a detailed study on the occurring host‐guest interactions and the nature of the energy transfer will be of fundamental interest.

## 4 Experimental Section/Methods

### 4.1 Materials and Methods

With the exception of **11‐DTCz‐BP**, which was synthesized using the method described in Section 4.3, all reagents and solvents were obtained from commercial sources and used without any further purifying. Terephthalic acid (H_2_bdc, 99 %) and dichloromethane (DCM) were purchased by Acros Organics. *N*, *N*‐dimethylformamide (DMF, ≥  99.9 %, anhydrous) was obtained from VWR In(NO_3_)_3_·xH_2_O (99.99 %) was supplied by Alfa Aesar.

### 4.2 Synthesis of MIL‐68(In)

#### 4.2.1 Synthesis of MIL‐68(In) as a Powder

The particle synthesis was adapted from Hosseini Monjezi et al. [42]. which was a modified solvothermal method involving N,N‐dimethylformamide (DMF) as the main solvent. In a typical synthesis procedure, 0.18 mmol of indium nitrate (In(NO_3_)_3_·7H_2_O, 77 mg), and 0.18 mmol of terephthalic acid (H_2_bdc, 30 mg) were dissolved in 9 mL of DMF, in a Teflon liner with a total volume of 25 mL. After adding 132 µL pyridine (1.62 mmol) as a modulator and stirring for 15 min, the Teflon liner was sealed in a stainless‐steel Paar autoclave and kept at 100°C for 24 h in an oven. The system was then allowed to cool naturally to room‐temperature. The resulting white powder was filtered off and washed several times with 10 mL of fresh DMF and 10 mL of ethanol, sequentially, then dried in a Leica EM CPD300 CO_2_ critical point dryer in order to remove the organic species encapsulated within the open MOF pores.

#### 4.2.2 Electrochemical Synthesis of MIL‐68(In) Thin Films

For the electrodeposition experiments, indium nitrate (In(NO_3_)_3_·7H_2_O) was first dissolved in DMF, and then benzene‐1,4‐dicarboxylic acid was added to the solution. The mixture was stirred vigorously to obtain a homogeneous solution. The MOF thin films were electrodeposited in a gold‐coated Si substrate using a DC power supply (Supporting Information). The electrochemical cell was equipped with two electrodes, a working electrode (thin film) and a counter electrode, which was operated at a constant voltage of 2 V throughout the entire time of the experiment for 20 min. It is important to note that no transformation occurs on the backside of the electrodes, and only the surfaces of the electrodes that face each other are electrochemically active. The thickness and uniformity of the film were controlled by adjusting the applied voltage and deposition time, allowing for precise control over the film's characteristics.

### 4.3 Synthesis of **11‐DTCz‐BP** Molecule

#### 4.3.1 11‐Bromodibenzo*[a,c]*Phenazine Synthesis

The synthesis was carried out according to a literature procedure [43]. A sealable vial was charged with phenanthrene‐9,10‐dione (500 mg, 2.40 mmol, 1.00 equiv.) and 4‐bromobenzene‐1,2‐diamine (449 mg, 2.40 mmol, 1.00 equiv.). Then, acetic acid (15.0 mL) was added, and the reaction mixture was stirred at 120°C for 15 h. The product mixture is then filtered and washed with water and ethanol. The product (832 mg, 2.32 mmol, 96% yield) was obtained as a yellow solid. The characterization of 11‐bromodibenzo[*a*,*c*]phenazine is provided in Supporting Information.

#### 4.3.2 11‐(3,6‐Di‐tert‐Butyl‐9H‐Carbazol‐9‐yl)Dibenzo*[a,c]*Phenazine

A sealable vial was charged with 11‐bromophenanthro[9,10‐*b*]quinoxaline (200 mg, 557 µmol, 1.00 equiv.), 3,6‐di‐*tert*‐butyl‐9*H*‐carbazole (156 mg, 557 µmol, 1.00 equiv.), NaO*t*Bu (107 mg, 1.11 mmol, 2.00 equiv.) and Pd(OAc)_2_ (12.5 mg, 55.7 µmol, 0.10 equiv.). It was then sealed, evacuated, and backfilled with argon three times. Subsequently, toluene (5.00 mL, abs.) was added, and P(*t*Bu)_3_ (22.5 mg, 111 µL, 111 µmol, 1.00 m in toluene, 0.20 equiv.) was added dropwise while stirring. The reaction mixture was stirred at 120°C for 64 h. The crude mixture was pulled over a Celite plug with dichloromethane. Then, all volatile components were removed under reduced pressure, and the crude product was further purified employing column chromatography (cyclohexane: ethyl acetate = 50:1 → 5:1). The product (309 mg, 554 µmol, 100% yield) was obtained as a bright yellow solid. The full characterizations of 11‐bromodibenzo[*a*,*c*]phenazine and 11‐(3,6‐di‐*tert*‐butyl‐9*H*‐carbazol‐9‐yl)dibenzo[*a*,*c*]phenazine are provided in Supporting Information.

### 4.4 Loading **11‐DTCz‐BP** MIL‐68(In) and MIL‐68(In) Thin Film

In this work, **11‐DTCz‐BP** was loaded into MIL‐68(In) pores, and the effects of dye loading on the physicochemical properties of the resulting composite were investigated. Generally, in a two‐step synthesis method, the pristine MOF was synthesized first and then immersed in **11‐DTCz‐BP** solution. In a typical procedure, a vial was filled with different weight percentages of the **11‐DTCz‐BP** molecule and 10 mL of dichloromethane (DCM) as an organic solvent. The mixture was stirred for 30 min at room‐temperature. Following that, 0.1 g of MIL‐68(In), previously activated with a CO_2_ critical point dryer to remove any adsorbed impurities and moisture, was weighed in a separate vial. Then, the solution containing **11‐DTCz‐BP** was added to the MIL‐68(In) vial. The mixture was sealed and left at room‐temperature for three days. After removing the cap, the mixture was dried at room‐temperature. Each sample was then placed in a desiccator for storage and further characterization.

### 4.5 Modeling the MIL‐68(In) Structures by Loading 11‐DTCz‐BP

The structure of MIL‐68(In) (cif entry 691619, Refocde LOQLEJ [35]) was freed from solvent molecules and geometry‐optimized with Quantum Espresso [44] using the Broyden‐Fletcher‐Goldfarb‐Shanno (BFGS) scheme. Atoms were described with ultrasoft Rappe‐Rabe‐Kaxiras‐Joannopoulos (RRKJ)‐type pseudopotentials [45]. Periodic plane‐wave DFT computations were performed using the generalized gradient approximation (GGA) with Perdew–Burke–Enzerhof (PBE) exchange correlation [46]. Calculations were restricted to the Γ‐point with an energy cut‐off of 40 Rydberg and a charge cut‐off of 400 Rydberg. To account for dispersion effects, the semi‐empirical Grimme D3‐correction scheme was adopted [47].

The optimized unit cell was further processed with Quantum Espresso. To fit **11‐DTCz‐BP**, the unit cell was duplicated along the z‐axis, forming a 1 × 1 × 3 supercell. Two **11‐DTCz‐BP** molecules were placed within the hexagonal channels of MIL‐68(In). The resulting supercell was allowed to freely relax.

### 4.6 Fluorescence Spectroscopy

Photoluminescence excitation and emission spectra, as well as the quantum yield determinations, were carried out on a Jobin Yvon Fluorolog 3 (Horiba) spectrometer using FluorEssence software. The device is equipped with a 450 W Xe short‐arc lamp (USHIO), double‐grated excitation and emission monochromators, and a photomultiplier tube (R928).

For the measurement of photoluminescence spectra, the powder samples were filled into round quartz glass cuvettes, while the thin films were fixed on a sample holder. Emission and excitation spectra were corrected for the spectral response of monochromators and detectors by the use of spectral corrections provided by the manufacturer. Additionally, excitation spectra were corrected by the spectral response of the lamp with a photodiode reference detector. An edge filter with a cutoff wavelength of 395 nm (Newport) was used for the thin films.

Photoluminescence quantum yields were measured with the device mentioned above in combination with a Horiba Quanta‐φ Integrating Sphere (F‐3029). The powders were filled into Micro Cell Cuvettes (18‐F/ST/C/Q/10) from Starna. Magnesium oxide was used as a reference material. Calibration of the Quanta‐φ Integrating sphere was checked by an additional standard (sodium salicylate powder, *λ*_ex_  =  340 nm, *λ*_em_ = 365‐600 nm; measured QY = 53.4 %, literature value: 53%) [48].

Photoluminescence lifetimes and fluorescence spectra were recorded with a second Fluorolog 3 additionally equipped with a UV xenon flashlamp (Exelitas FX‐1102) and a TCSPC (time‐correlated single‐photon counting) upgrade using DataStation software. Samples were excited with a diode (Horiba DD‐287) or a laser (Horiba DD‐375L). Emission intensity decay was fitted with a biexponential decay function using Decay Analysis Software 6. Phosphorescence spectra were measured using the UV xenon flashlamp and FluorEssence software.

### 4.7 X‐Ray Diffraction

XRD patterns were obtained by a Bruker D8 ADVANCE AXS diffractometer with Cu Kα radiation (λ = 1.5418 Å) operating at 40 kV and 40 mA in θ–θ geometry, equipped with a LynxEye detector. The samples were investigated with a scan speed of 1 s and an increment of 0.02° per step.

### 4.8 Scanning Electron Microscopy

SEM was performed using a FEI Philips XL 30 Field Emission Gun Environmental Scanning Electron Microscope (FEG‐ESEM) (FEI Co., Hillsboro, OR, USA) to investigate the morphologies, crystallinity, and microstructures of MIL‐68(In) and **11‐DTCz‐BP**@MIL‐68(In). Prior to measurement, the samples were sputtered with an Au–Pd target (4–5 nm thickness) using a Bal‐Tec MCS 010 coating system to avoid charging effects by increasing surface conductivity. The measurements were carried out in a high‐vacuum (1.5 Torr) using a 20 keV acceleration voltage.

### 4.9 Thermogravimetric Analysis

TGA of the MIL‐68(In) and **11‐DTCz‐BP**@MIL‐68(In) samples was executed using a Mettler Toledo TGA2 Star system in a pinhole aluminum crucible with a heating ramp rate of 10°C/min under a nitrogen atmosphere to 1000°C.

### 4.10 Fourier Transform Attenuated Total Reflection Infrared Spectroscopy

FT‐ATR‐IR was performed using a Bruker Alpha FTIR. To collect FTIR spectral data of synthesized MIL‐68(In) before and after **11‐DTCz‐BP** immobilization process, first baseline calibration measurement on a background was performed for 10 scans. For each sample, an average of 130 scans was recorded at a resolution setting of 4 cm^−1^ in the range of 400–4000 cm^−1^ with background subtraction.

### 4.11 UV–vis Spectroscopy

UV–vis absorption spectra were collected by using an Agilent Cary 5000 UV–vis spectrophotometer under ambient conditions.

## Conflicts of Interest

The authors declare no conflict of interest.

## Supporting information




**Supporting file**: advs74832‐sup‐0001‐SuppMat.docx

## Data Availability

The data that support the findings of this study are available in the supplementary material of this article. The research data supporting this publication can be accessed at https://doi.org/10.17630/43999364‐fb89‐4aa9‐920b‐839f4c6ab641
