# Supplementary material for: Enhanced Energy Transfer from a Metal–Organic Framework to a Highly Confined Organic Phosphorescent Dye
Source: Adv Sci (Weinh). 2026 Mar 15;13(30):e00068. doi: 10.1002/advs.202600068 (PMC13248832; doi:10.1002/advs.202600068)
Supplement: Supplementary file 1 — Supporting file: advs74832‐sup‐0001‐SuppMat.docx [file ADVS-13-e00068-s001.docx]

**Supporting Information**

**Enhanced Energy Transfer from a Metal-Organic Framework to a Highly Confined Organic Phosphorescent Dye**

*Bahram Hosseini Monjezi, Thomas Kasper, Gloria Hong, Changfeng Si, Robert Oestreich, Oliver Weingart, Peter G. Weidler, Christof Wöll, Eli Zysman-Colman, Christoph Janiak, Stefan Bräse, Klaus Müller-Buschbaum, Alexander Knebel**

*Corresponding author: [alexander.knebel@uni-jena.de](mailto:alexander.knebel@uni-jena.de)

**Contents**

[1. General Information on Analysis Methods and 11-DTCz-BP Synthesis 5](#_Toc203559455)

[1.1 Materials and Methods 5](#_Toc203559456)

[1.2 Theoretical Calculation: 5](#_Toc203559457)

[1.3 Reaction Monitoring 5](#_Toc203559458)

[1.4 Infrared Spectroscopy (IR) 5](#_Toc203559459)

[1.5 Mass Spectrometry (MS) 5](#_Toc203559460)

[1.6 Melting Point (m.p.) 6](#_Toc203559461)

[1.7 Thermogravimetric analysis (TGA) 6](#_Toc203559462)

[1.8 Nuclear Magnetic Resonance Spectroscopy (NMR) 7](#_Toc203559463)

[2. Synthesis Procedure of MIL-68(In) thin films and 11-DTCz-BP 7](#_Toc203559464)

[2.1 Electrochemical synthesis of MIL-68(In) thin films 7](#_Toc203559465)

[2.2 Synthesis procedure of 11-DTCz-BP (3) 8](#_Toc203559466)

[2.3 characterization of 11-Bromodibenzo[a,c]phenazine: 8](#_Toc203559467)

[2.4 Characterization of 11-(3,6-Di-tert-butyl-9H-carbazol-9-yl)dibenzo[a,c]phenazine: 9](#_Toc203559468)

[2.5 UV-Vis Absorption and Photophysical Properties of 11-DTCz-BP 11](#_Toc203559469)

[3. References 14](#_Toc203559470)

**Table S 1** Photoluminescence lifetimes of MIL-68(In), **11-DTCz-BP** and **11-DTCz-BP** @MIL-68(In) as a powder and thin film at room temperature and 77 K.

| Sample | Temperature | ***λ*_ex_**  [nm] | ***λ*_em_**  [nm] | ***τ*_1_**  [ns] | ***τ*_2_**  [ns] | ***τ*_phos/11-DTCz-BP_**  [µs] |
| --- | --- | --- | --- | --- | --- | --- |
| MIL-68(In) | RT | 287 | 354 | 0.79(2)^a^ | 2.67(2) | - |
|  | 77 K | 287 | 341 | 0.43(5)^a^ | 2.23(2) | - |
| **11-DTCz-BP** | RT | 368 | 507 | 2.43(2) | 12.6(3) | - |
|  | 77 K | 368 | 494 | 3.45(2) | 7.14(2) | - |
|  | 77 K | 368 | 508 | 3.72(2) | 8.35(3) | - |
|  | 77 K | 360 | 627 | - | - | 335(5) |
| **11-DTCz-BP** @MIL-68(In) | RT | 287 | 360 | 0.95(1)^a^ | 3.07(1) | - |
|  | RT | 287 | 507 | 5.59(5) | 15.97(5) | - |
|  | RT | 250 | 507 | - | - | 130(1) |
|  | RT | 368^b^ | 507 | 4.93(3) | 13.46(2) | - |
|  | 77 K | 287 | 360 | 1.00(3)^a^ | 2.91(2) | - |
|  | 77 K | 287 | 508 | 6.5(3) | 19.4(7) | - |
|  | 77 K | 368^b^ | 508 | 5.47(4) | 13.87(2) | - |
| MIL-68(In) on gold substrate | RT | 287 | 442 | 2.53(3) | 8.94(4) | - |
|  | 77 K | 287 | 324 | 1.81(3) | 9.8(3) | - |
|  |  | 287 | 443 | 3.59(4) | 10.35(6) | - |
| **11-DTCz-BP** @MIL-68(In) on gold substrate | RT | 287 | 508 | 3.73(5) | 9.73(8) | - |
|  | RT | 250 | 508 | - | - | 7.5(3) |
|  | 77 K | 287 | 506 | 5.54(8) | 17.9(6) | - |

^a^Pulse width of diode used for excitation

^b^Direct excitation of **11-DTCz-BP** in composite

**Table S 2** Photoluminescence quantum yields of MIL-68(In), **11-DTCz-BP** and **11-DTCz-BP**@MIL-68(In)

| Sample | ***λ*_ex_**  [nm] | ***λ*_em_**  [nm] | PLQY  [%] |
| --- | --- | --- | --- |
| MIL-68(In) | 318 | 334-600 | 3.3(3) |
| **11-DTCz-BP** | 362 | 450-707 | 9.0(5) |
| **11-DTCz-BP** @MIL-68(In) | 322 | 450-608 | 13.8(9) |

# **1. General Information on Analysis Methods and 11-DTCz-BP Synthesis**

- 1. **Materials and Methods**

Solvents, reagents, and chemicals were purchased from ABCR, Chempur, Carl Roth, Sigma Aldrich, and Fisher Scientific and were used without further purification. Air- and moisture-sensitive reactions were carried out under argon atmosphere in sealable vials or flame-dried flasks using standard Schlenk techniques. Liquids were added with a stainless-steel cannula, and solids were added in a powdered shape. Solvents were evaporated under reduced pressure at 45 °C using a rotary evaporator. For solvent mixtures, each solvent was measured volumetrically. Column chromatography was performed using Merck silica gel 60 (0.040 × 0.063 mm, 230–400 mesh ASTM) and quartz sand (glowed and purified with hydrochloric acid).

- 1. **Theoretical Calculation: Density functional theoretical (DFT)**

Calculation and time-dependent density functional theoretical (TD-DFT) calculations were performed using Gaussian 09 Revision D.01 software^[1]^ in the gas phase. The ground state geometries were optimized employing the PBE0^[2]^ functional with the Pople 6-31G(d,p) basis set,^[3]^ in the gas phase. Transitions to excited singlet states and triplet states were calculated using TD-DFT within the Tamm-Dancoff approximation (TDA) based on the optimized ground-state geometries.^[4]^ Molecular orbitals were visualized using GaussView 6.0 software.^[5]^

- 1. **Reaction Monitoring**

Routine monitoring of reactions was performed using silica gel coated aluminum plates (Merck, silica gel 60, F254) analyzed under UV light at 254 and 365 nm. Solvent mixtures are understood as v/v.

- 1. **Infrared Spectroscopy (IR)**

The infrared spectra were recorded with a Bruker, Alpha P instrument. All samples were measured by attenuated total reflection (ATR). The positions of the absorption bands are given in wavenumbers ῡ in cm^–1^ and were measured in the range from 3600 cm^–1^ to 500 cm^–1^. Characterization of the absorption bands was done in dependence on the transmission strength with the following abbreviations: vs (very strong, 100−90%), s (strong, 89−70%), m (medium, 59−40%), w (weak, 39−10%), vw (very weak, 0−9%).

- 1. **Mass Spectrometry (MS)**

Fast atom bombardment (FAB) experiments were conducted using a Finnigan MAT 90 (70 eV) instrument, with 3-nitrobenzyl alcohol (3-NBA) as matrix and reference for high resolution. For the interpretation of the spectra, molecular peaks [M]+, peaks of protonated molecules [M+H]+, and characteristic fragment peaks are indicated with their mass-to-charge ratio (m/z) and their intensity in percent, relative to the base peak (100%) is given. In the case of high-resolution measurements, the tolerated error is 0.0005 m/z.

- 1. **Melting Point (m.p**.)

Melting points were measured using an OptiMelt MPA100 device by Stanford Research System.

- 1. **Thermogravimetric analysis (TGA)**

Thermogravimetric analysis (TGA) of MIL-68(In) and 11-DTCz-BP (3)@MIL-68(In) was performed from 20-1000 °C under nitrogen gas flow to assess the thermal stability, fractional weight loss of volatile components and the amount of incorporated 11-DTCz-BP (3) molecule in the pore channels by determining the residues weights (Figure S1). The weight loss curves of MIL-68(In) and 11-DTCz-BP (3)@MIL-68(In) exhibit almost similar thermal stability, and both were stable over 400 °C with intact 91 and 95 % weight, respectively. The first stage of weight loss for these two samples is attributed to thermal desorption of residual solvents such as water or DCM, remaining in the smaller 5 Å pore channels, or atmospheric gas desorption, whereas the most significant weight loss occurs in the temperature range of 400–900 °C. At temperatures above 400 °C, the MIL-68(In) starts to decompose and its framework experienced a mass loss of 47.5 wt.-%, which could be attributed to carbonization of organic compounds, and the remaining residues (42.7 wt.-%) represented metallic oxides (Indium oxide) and ashes.[32] In the end, 11-DTCz-BP (3)@MIL68(In) has fewer residues (30.1 wt.-%) than MIL68(In) (42.7 wt.-%), implying less Indium oxide. On the basis of this observation, the wt.-% loading of the dye was estimated to be 12 wt.-%.


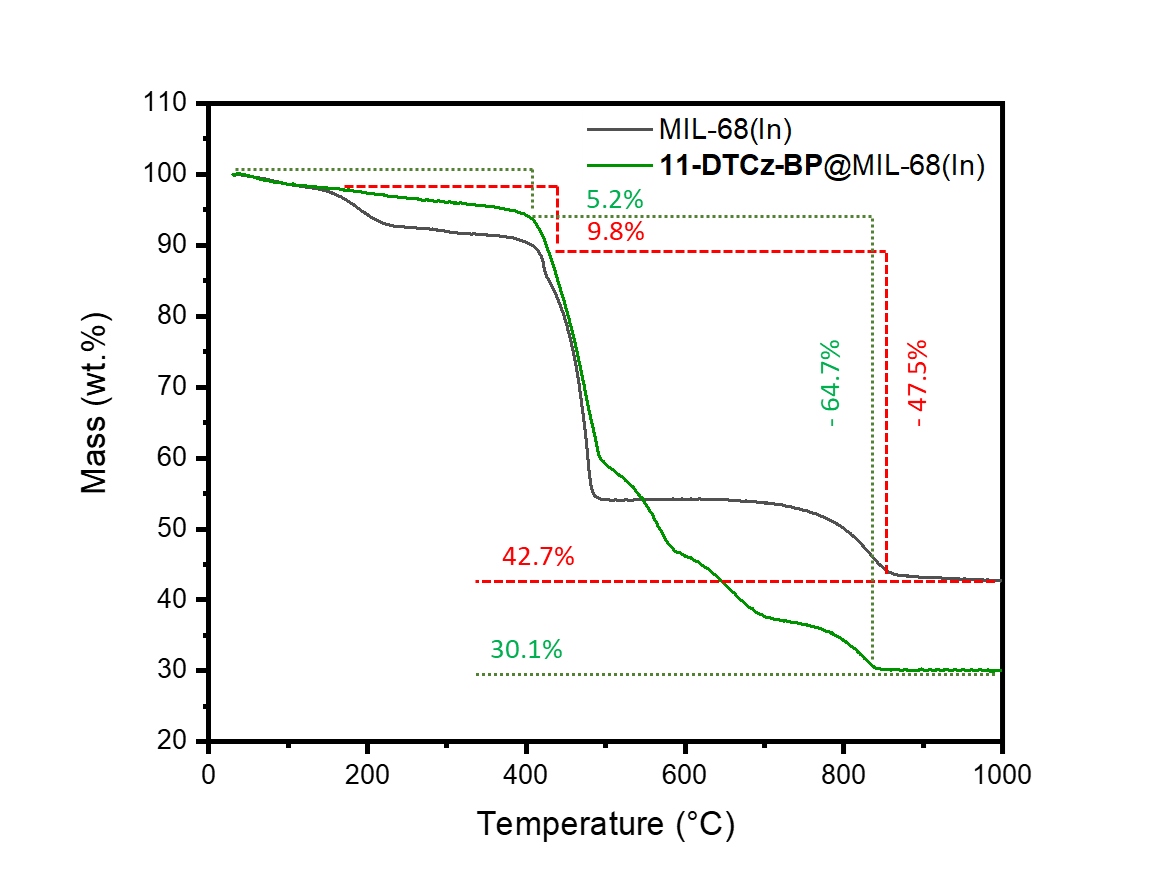


**Figure S1** Thermogravimetric analysis (TGA) of MIL-68(In) and 11-DTCz-BP (3)@MIL-68(In) at a heating rate of 10 °C/min under a nitrogen atmosphere.

- 1. **Nuclear Magnetic Resonance Spectroscopy (NMR)**

^1^H NMR spectra were recorded on Bruker Avance 400 (400 MHz) spectrometer. Chemical shifts are given in parts per million (δ/ppm), downfield from tetramethylsilane (TMS), and are referenced to chloroform (7.26 ppm) as internal standard. All coupling constants are absolute values, and *J* values are expressed in Hertz (Hz). The description of signals includes: s = singlet, bs = broad singlet, d = doublet, t = triplet, dd = doublet of doublets, ddd = doublet of doublet of doublets, dt = doublet of triplets, q = quartet, quin = quintet, sxt = sextet, sept = septet, m = multiplet. The spectra were analyzed according to the first order. ^13^C NMR spectra were recorded on Bruker Avance 400 (100 MHz) spectrometer. Chemical shifts are expressed in parts per million (δ/ppm) downfield from tetramethylsilane (TMS) and are referenced to chloroform (77.16 ppm) as internal standard.

# **2.** **Synthesis Procedure of MIL-68(In) thin films and 11-DTCz-BP**

**2.1 Electrochemical synthesis of** **MIL-68(In) thin films**

Figure S2 presents a schematic of the electrochemical deposition setup used to create MIL-68(In) thin films. It illustrates the two-electrode configuration, with gold-coated silicon substrates serving as the working and counter electrodes, and includes the solution of In(NO₃)₃ and BDC where the deposition occurs. The MIL-68(In) thin films formed through this process feature one-dimensional pore channels that allow for the incorporation of the 11-DTz-1BP dye, leading to a well-defined crystallographic confinement within the pores.

*
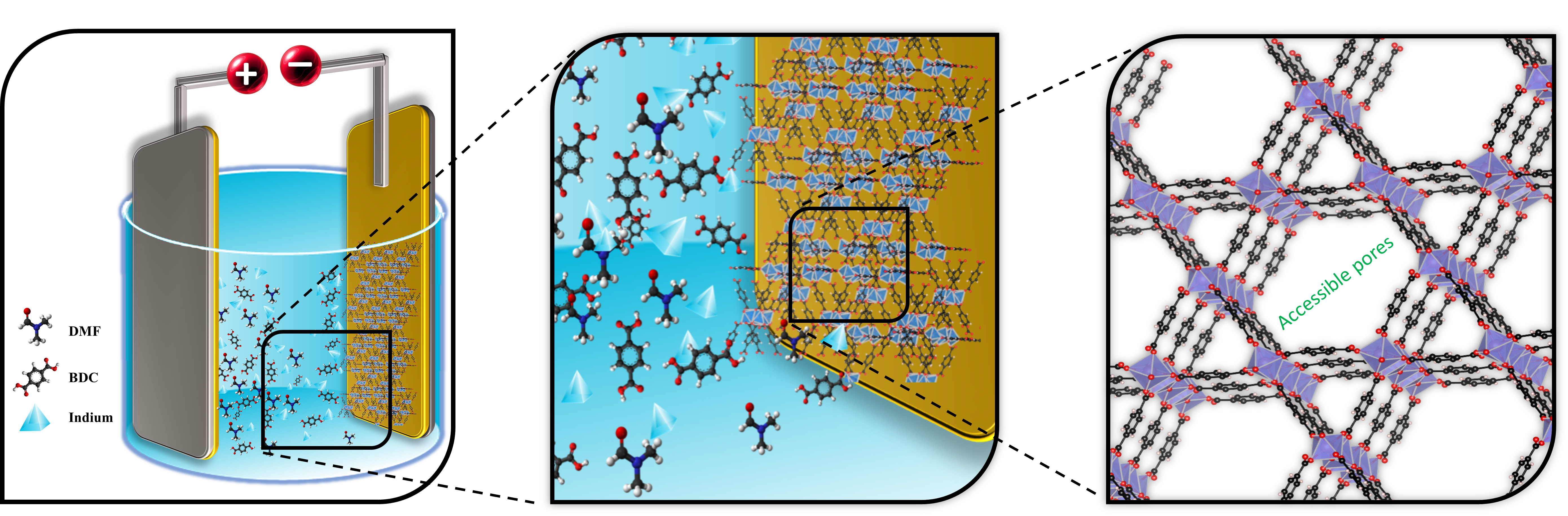
*

**Figure S 2** Schematic of the electrochemical setup for MIL-68(In) thin film deposition on gold-coated Si substrates at 2 V for 20 minutes.

**2.2** **Synthesis procedure of 11-DTCz-BP (3)**

The designed molecule was synthesized as outlined in Scheme 1 and the chemical characterization is provided below. 11-Bromodibenzo[a,c]phenazine was obtained from a literature-known procedure^[6]^ and subsequently used in a Buchwald- Hartwig amination reaction with 3,6-di-tert-butyl-carbazole (DTCz, 2) to obtain the D-A-type emitter 11-DTCz-BP (3) as a yellow solid with a yield of 91%.

**Scheme S 1.** Synthesis of **11-DTCz-BP** (**3**) via Buchwald-Hartwig amination.

**2.3 characterization of 11-Bromodibenzo[a,c]phenazine:**

The characterizations of synthesized 11-bromodibenzo[*a*,*c*]phenazine can be found below.

**^1^H NMR** (400 MHz, CDCl_3_) δ = 9.39–9.32 (m, 2H, 7-H, 17-H), 8.56 (d, *^3^J* = 8.1 Hz, 2H, 10-H, 13-H), 8.51 (d, *^4^J* = 2.2 Hz, 1H, 20-H), 8.18 (d, *^3^J* = 8.9 Hz, 1H, 3-H), 7.91 (dd, *^3^J* = 8.9 Hz, *^4^J* = 2.2 Hz, 1H, 2-H), 7.81 (t, *^3^J* = 7.5 Hz, 2H, 9-H, 13-H), 7.75 (t, *^3^J* = 7.5 Hz, 2H, 8-H, 15-H) ppm. **HRMS–FAB** *(m/z)*: Calc. for [C_20_H_12_N_2_^79^Br]^+^: 359.0178; found: 359.0177. **R***_f_* = 0.67 (Cyclohexane : ethyl acetate = 4:1). **M.p.:** 246-252 °C.

**2.4 Characterization of** **11-(3,6-Di-tert-butyl-9H-carbazol-9-yl)dibenzo[a,c]phenazine:**

The characterizations of synthesized 11-(3,6-di-*tert*-butyl-9*H*-carbazol-9-yl)dibenzo[*a*,*c*]phenazine can be found below.

**^1^H NMR** (400 MHz, CDCl_3_) δ = 9.45 (dd, *^3^J* = 7.8 Hz, *^4^J* = 1.7 Hz, 1H, 12-H), 9.42 (dt, *^3^J* = 8.0 Hz, *^4^J* = 1.5 Hz, 1H, 9-H), 8.60 (d, *^3^J* = 8.0 Hz, 2H, 6-H, 15-H), 8.55–8.48 (m, 2H, 2-H, 19-H), 8.20 (d, *^4^J* = 1.8 Hz, 2H, 5'-H), 8.12 (dt, *^3^J* = 9.0 Hz, *^4^J* = 1.8 Hz, 1H, 20-H), 7.88–7.80 (m, 2H, 7-H, 14-H), 7.79–7.73 (m, 2H, 8-H, 13-H), 7.66–7.59 (m, 2H, 2'-H), 7.54 (dd, *^3^J* = 8.7 Hz, *^4^J* = 1.8 Hz, 2H, 3'-H), 1.50 (s, 18H, C*H*_3_) ppm. **^13^C NMR** (101 MHz, CDCl_3_) δ = 143.87 (2C, C-4'), 143.22 (C-17), 143.02 (C-1), 142.64 (C-4), 141.04 (C-18), 139.55 (C-3), 139.04 (2C, C-1'), 132.42 (C-16), 132.23 (C-5), 131.07 (C-19), 130.72 (C-14), 130.56 (C-7), 130.44 (C-11), 130.32 (C-10), 128.94 (C-20), 128.19 (2C, C-8, C-13), 126.61 (C-9), 126.43 (C-12), 125.06 (C-2), 124.13 (2C, C-6'), 124.10 (2C, C-3'), 123.15 (2C, C-6, C-15), 116.59 (2C, C-5'), 109.58 (2C, C-2'), 34.97 (2C, C_q_), 32.16 (6C, *C*H_3_) ppm. **MS** (FAB, 3-NBA), m/z (%): 558 (24) [M+H]^+^, 557 (24) [M]^+^. **HRMS–FAB** *(m/z)*: Calc. for [C_40_H_35_N_3_]^+^: 557.2825; found: 557.2825. **IR** (ATR, ṽ) = 3060 (w), 2952 (vs), 2921 (vs), 2854 (s), 1615 (m), 1581 (w), 1499 (vs), 1490 (s), 1476 (vs), 1449 (vs), 1357 (vs), 1312 (vs), 1299 (vs), 1293 (s), 1259 (s), 1235 (s), 1200 (s), 1176 (m), 1037 (s), 877 (s), 841 (m), 813 (vs), 807 (vs), 764 (vs), 724 (vs), 611 (vs), 554 (vs), 439 (s), 424 (m) cm^–1^. **R***_f_* = 0.73 (Cyclohexane : ethyl acetate = 4:1). **M.p.:** 267-272 °C.


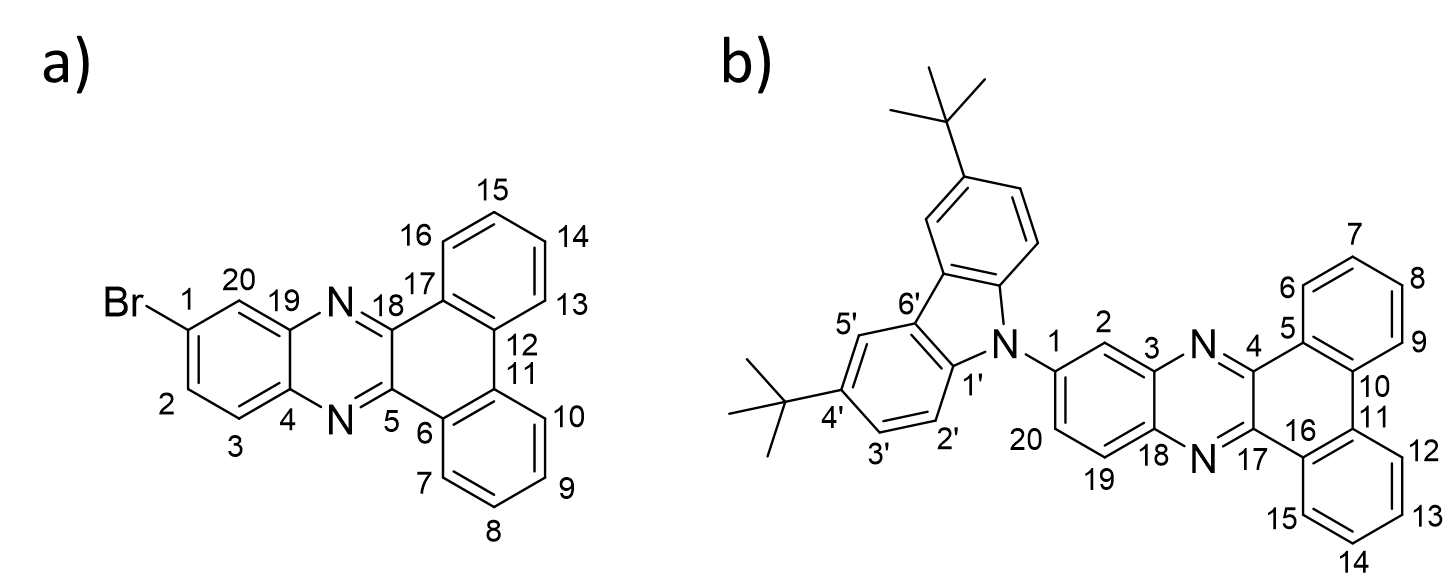


**Figure S 3.** Chemical structure of a) 11-bromodibenzo[*a*,*c*]phenazine and b) 11-(3,6-di-*tert*-butyl-9*H*-carbazol-9-yl)dibenzo[*a*,*c*]phenazine.

**Figure S 4.** ^1^H NMR of 11-bromodibenzo[*a*,*c*]phenazine.

**Figure S 5.** ^1^H NMR of 11-(3,6-di-*tert*-butyl-9*H*-carbazol-9-yl)dibenzo[*a*,*c*]phenazine.

**Figure S 6.** ^13^C NMR of 11-(3,6-di-*tert*-butyl-9*H*-carbazol-9-yl)dibenzo[*a*,*c*]phenazine.

**2.5 UV-Vis Absorption and Photophysical Properties of 11-DTCz-BP**

The results of UV–Vis absorption spectra of 11-DTCz-BP at room temperature in diluted DCM solution revealed that only the intensity of the absorption peaks increased proportionally with the dye concentration, while the position of the maximum absorption peak remained unchanged, without any noticeable redshift (Figure S7 a). A redshift would indicate a deviation from Lambert–Beer’s law, suggesting possible dye aggregation in solution. However, in this case, no such shift was observed, implying the absence of aggregation in the concentration range of 1 to 10 µM.

Figure S7 b shows the photoluminescence excitation (PLE) spectra of 11-DTCz-BP at different emission wavelengths (550, 600, and 700 nm), while Figure S7 c presents the corresponding emission spectra recorded near the absorption and excitation maxima in the visible region at 450 and 475 nm.


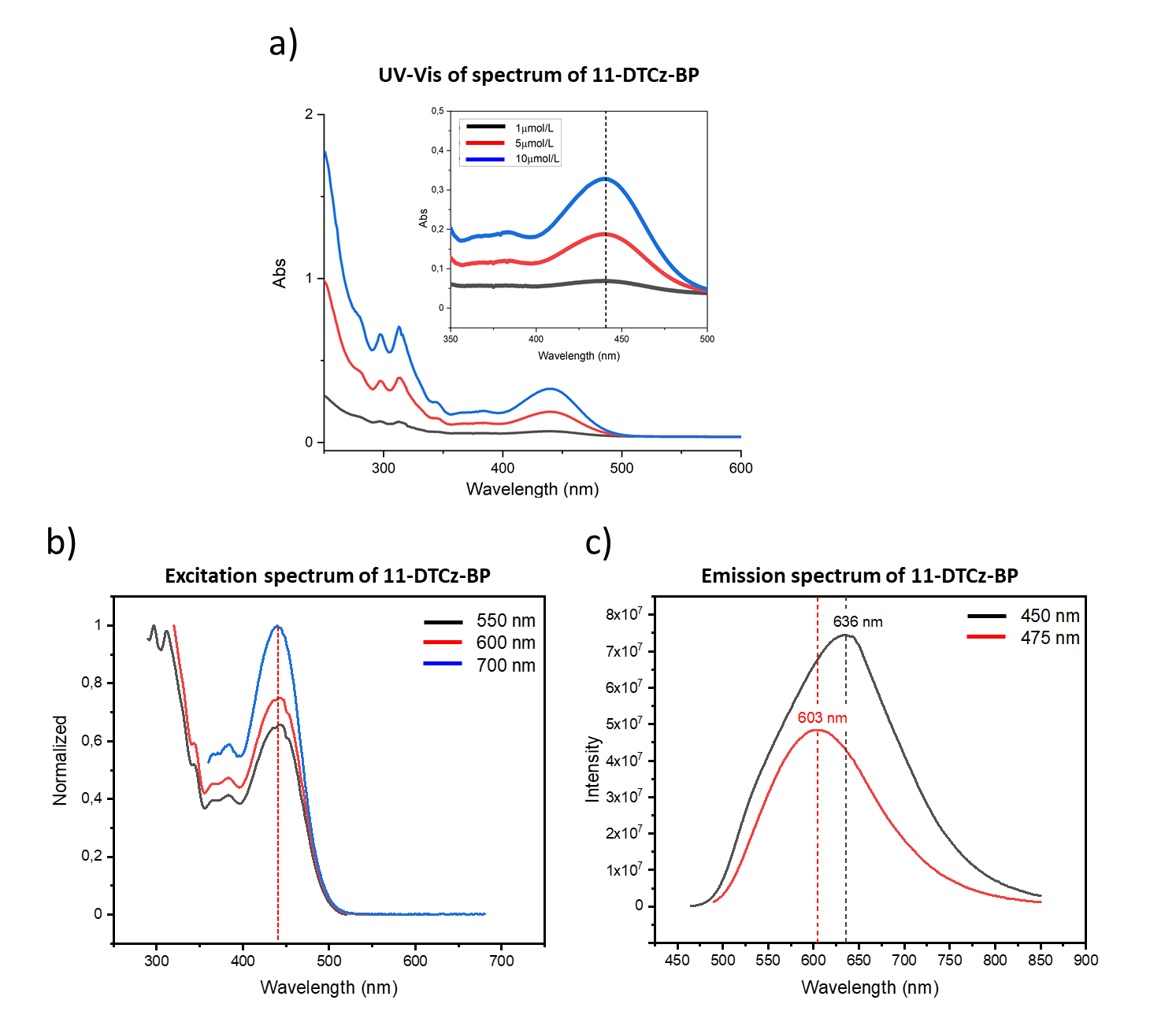


**Figure S 7** UV-Vis absorption spectra of **11-DTCz-BP** at concentrations of 1, 5, and 10 µmol/L in DCM, showing no evidence of aggregation. (b) Excitation spectra of **11-DTCz-BP** at emission wavelengths of 550, 600, and 700 nm. (c) Emission spectra recorded near the absorption and excitation maxima in the visible region, at 450 and 475 nm, in DCM.


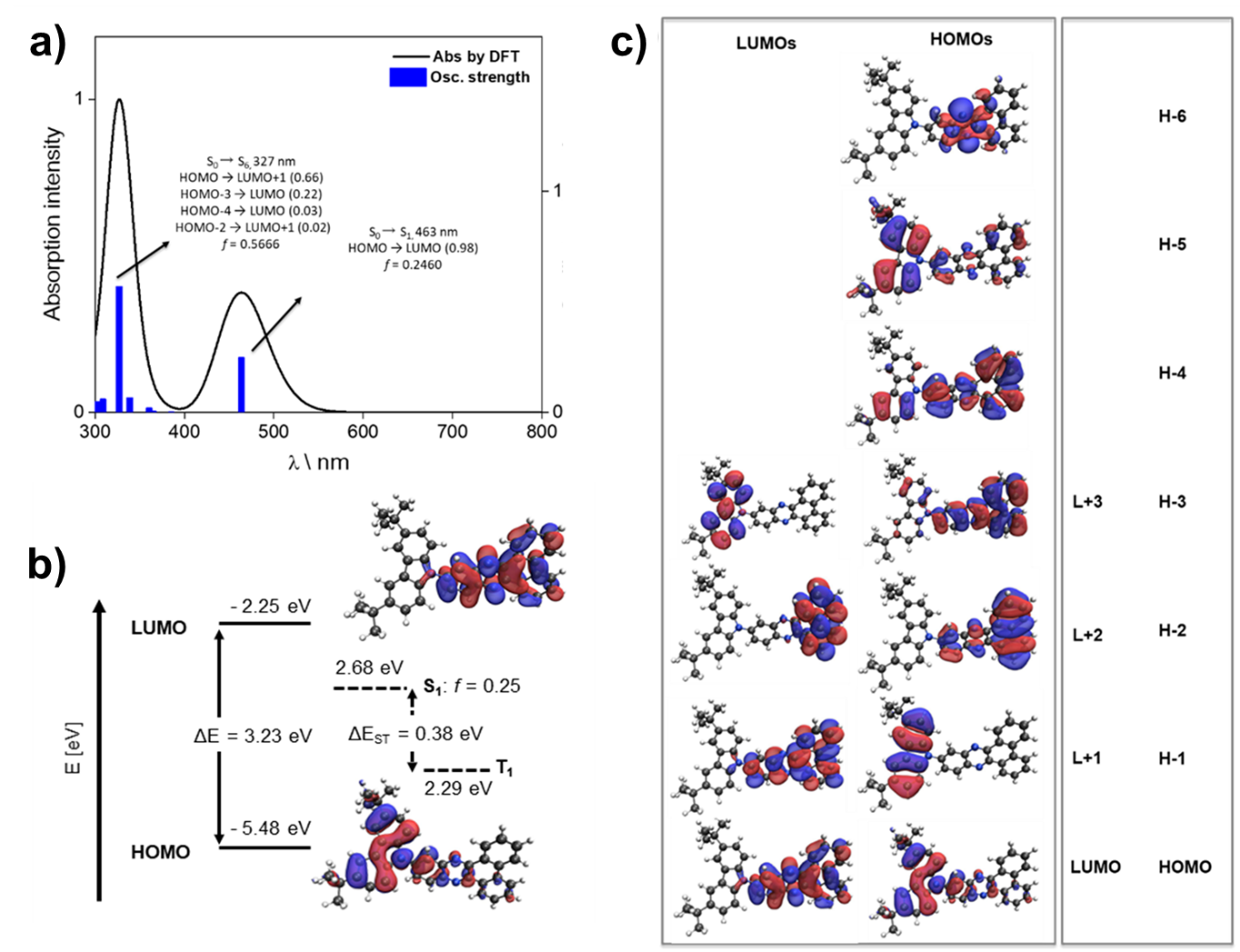


**Figure S 8** a) TDA-DFT simulation and experimental UV-Vis absorption spectra of **11-DTCz-BP**  (fitted FWHM = 3000 cm^-1^); b) Geometry optimization and excited state (TD)-DFT calculation results for 11-DTCz-BP; Theoretical modelling of the energies of the HOMO/LUMO orbitals and the S_1_ and T_1_ states. Computation in the gas phase using the PBE0/6-31G(d,p). c) Calculated distribution of molecular orbitals for **11-DTCz-BP**. (isovalue= 0.02)





**Figure S 9** Simulated powder X-ray diffraction (PXRD) patterns of pristine MIL-68(In) (bottom) and the proposed host–guest model of 11-DTCz-BP fully occupying the 1D channels of MIL-68(In) (top), generated from the corresponding structural models (.cif files).

# **3. References**

[1] M. J. Frisch, G. W. Trucks, H. B. Schlegel, G. E. Scuseria, M. A. Robb, J. R. Cheeseman, G. Scalmani, V. Barone, G. A. Petersson, H. Nakatsuji, X. Li, M. Caricato, A. V. Marenich, J. Bloino, B. G. Janesko, R. Gomperts, B. Mennucci, H. P. Hratchian, J. V. Ortiz, A. F. Izmaylov, J. L. Sonnenberg, Williams, F. Ding, F. Lipparini, F. Egidi, J. Goings, B. Peng, A. Petrone, T. Henderson, D. Ranasinghe, V. G. Zakrzewski, J. Gao, N. Rega, G. Zheng, W. Liang, M. Hada, M. Ehara, K. Toyota, R. Fukuda, J. Hasegawa, M. Ishida, T. Nakajima, Y. Honda, O. Kitao, H. Nakai, T. Vreven, K. Throssell, J. A. Montgomery Jr., J. E. Peralta, F. Ogliaro, M. J. Bearpark, J. J. Heyd, E. N. Brothers, K. N. Kudin, V. N. Staroverov, T. A. Keith, R. Kobayashi, J. Normand, K. Raghavachari, A. P. Rendell, J. C. Burant, S. S. Iyengar, J. Tomasi, M. Cossi, J. M. Millam, M. Klene, C. Adamo, R. Cammi, J. W. Ochterski, R. L. Martin, K. Morokuma, O. Farkas, J. B. Foresman, D. J. Fox, Wallingford, CT ***2016***.

[2] C. Adamo, V. Barone, *J. Chem. Phys.* **1999**, *110*, 6158.

[3] S. Grimme, *Chem. Phys. Lett.* **1996**, *259*, 128.

[4] S. Hirata, M. Head-Gordon, *Chem. Phys. Lett.* **1999**, *314*, 291.

[5] R Dennington, T. Keith and J. Millam GaussView, Version 6.1.1, KS: Semichem Inc.: Shawnee Mission, 2019.

[6] C. Zhou, S. Xiao, M. Wang, W. Jiang, H. Liu, S. Zhang, B. Yang, *Frontiers in chemistry* **2019**, *7*, 141.
